# Dual calcium-voltage optical mapping of regional voltage and calcium signals in intact murine *RyR2*-R2474S hearts

**DOI:** 10.1016/j.jmccpl.2024.100121

**Published:** 2024-12

**Authors:** Yangpeng Li, Zhu Liu, Christopher O'Shea, Jianhong Li, Xian Luo, Tangting Chen, Xianhong Ou, Weichao Liu, Guoliang Hao, Christopher L.-H. Huang, Davor Pavlovic, Xiaoqiu Tan, Ming Lei

**Affiliations:** a Key Laboratory of Medical Electrophysiology of the Ministry of Education, Medical Electrophysiological Key Laboratory of Sichuan Province, Institute of Cardiovascular Research, Southwest Medical University, Luzhou, Sichuan 646000, China; b Department of Cardiology, the Affiliated Hospital of Southwest Medical University, Luzhou, Sichuan 646000, China; c Institute of Cardiovascular Sciences, University of Birmingham, Birmingham B15 2TT, UK; d Henan SCOPE Research Institute of Electrophysiology Co. Ltd., Kaifeng 475000, China; e Physiological Laboratory, Department of Biochemistry, University of Cambridge, Cambridge CB2 3EG, UK; f Department of Pharmacology, University of Oxford, Mansfield Road, Oxford OX1 3QT, UK; g Department of Physiology, School of Basic Medical Sciences, Southwest Medical University, Luzhou, Sichuan 646000, China

**Keywords:** Catecholaminergic polymorphic ventricular tachycardia, Action potentials, Ca^2+^ transients, *RyR2*, Murine cardiac models

## Abstract

Abnormal regional variations in electrical and calcium homeostasis properties have been implicated in catecholaminergic polymorphic ventricular tachycardias (CPVT) attributable to abnormal RyR2-mediated store Ca^2+^ release, but their underlying mechanism have not been well explored in intact hearts.

**Methods:**

We performed in vivo and ex vivo studies including high throughput mapping of Ca^2+^ transients (CaT) and transmembrane voltage (V_m_) in murine wild-type (WT) and heterozygous *RyR2*-R2474S/+ hearts, before and during isoprenaline (ISO) challenge.

**Results:**

ISO-challenged *RyR2*-R2474S/+ showed increased incidence of arrhythmia accompanied by abnormal Ca^2+^ transients compared to WT. CaT duration (CaTD) in the LV apex amongst regions studied both before and during ISO challenge in both WT and *RyR2*-R2474S/+ ventricles. *RyR2*-R2474S/+ ventricles showed prolonged CaTD, both before and during isoprenaline (ISO) challenge. Conversely, action potential durations (APD) were the same in WT and RyR2-R2474S/+ ventricles and identically reduced by ISO challenge. *RyR2*-R2474S/+ showed *V*_m_-CaT latencies at time to half decay, but not rise time to peak, which were significantly prolonged compared to WT in all ventricular regions examined with ISO challenge. Following burst pacing, ventricular localized concordant alternans in CaT and APD were readily observed in *RyR2*-R2474S/+ but not in WT mice. Such CaT and APD alternans occurred mostly semiannually in specific regions of the ventricular pre-occurrence of VT.

**Conclusion:**

The pro-arrhythmic *RyR2*-R2474S/+ phenotype in intact hearts thus directly parallels delayed regional CaT recovery properties and alteration of *V*_m_-CaT latencies. Studies suggest that discordant localized calcium alternans are mechanistically responsible for action potential duration alternans and occurrence of VT in *RyR2*-R2474S/+ mice.

## Highlights

• Clarified the electrophysiological mechanisms in the RyR2-R2474S model using dual-dye optical mapping.
• Defined the disrupted coupling dynamics between transmembrane voltage and intracellular calcium based on C-clock and M-clock.
• Determined the role of variable CaT alternans in the susceptibility to ventricular arrhythmias in a CPVT mouse model

## Graphical abstract

**Figure:**
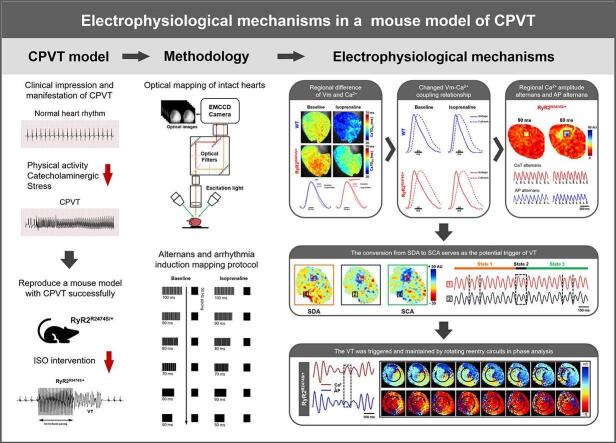

## 1 Introduction

Catecholaminergic polymorphic ventricular tachycardia (CPVT) is a rare genetic arrhythmic syndrome, which can progress to potentially fatal ventricular fibrillation (VF) triggered by physical activity, stress and catecholamine challenge [[1], [2], [3], [4]]. Symptoms typically include recurrent syncope, seizures, or sudden death following exertion or emotional stress [5]. Previous research has identified several genes associated with CPVT and sarcoplasmic reticular (SR) Ca^2+^ release, including *RYR2*, *FKBP12.6*, *CASQ2*, *TRDN*, and *CALM1*-*3*[[6], [7], [8], [9], [10], [11], [12]]. The occurrence of cardiomyocyte cytosolic Ca^2+^ waves is more likely when background cytosolic [Ca^2+^] increases due to RyR sensitization by catecholaminergic challenge.

The mechanisms by which defective RyR2 channels lead to ventricular arrhythmias in CPVT remain incompletely understood. For instance, cardiac alternans have been linked to ventricular arrhythmia in CPVT patients [13], with Ca^2+^ alternans believed to play a central role in their genesis. However, at the single-cell level, Ca^2+^ alternans can occur independently of action potential duration (APD) alternans. Wen et al. [14] demonstrated this dissociation by simultaneously recording membrane potential and Ca^2+^ transients in isolated cardiomyocytes, showing that Ca^2+^ alternans could occur without APD alternans, while APD alternans did not occur without Ca^2+^ alternans. A growing body of evidence supports the idea that Ca^2+^ dysregulation primarily contributes to cardiac alternans [15,16].

Over the past two decades, emerging evidence strongly suggests that the normal electrical function of the heart results from dynamic interactions between membrane ion channels and intracellular Ca^2+^ handling proteins within a complex molecular network [17,18]. Recently, a two-clock theory was proposed to explain normal and abnormal cardiac electrophysiological function [18]. According to this theory, a membrane (M) clock governs surface electrophysiological changes and excitation conduction, which in turn influences the calcium (C-) clock by modulating cytosolic Ca^2+^ levels. This triggers cycles of store Ca^2+^ release and re-uptake, primarily regulated by the M-clock's activation and recovery processes. However, the C-clock also influences the M-clock by altering action potential conduction, recovery, and post-recovery stability, as well as exerting longer-term effects on channel expression. Autonomic sympathetic and parasympathetic control mechanisms act at different control points within the C- and M-clocks, respectively. This novel clock theory holds promise for elucidating how dysfunction of specific membrane or Ca^2+^ handling proteins leads to cardiac arrhythmias, including CPVT.

In the present study, we studied hearts from the wild type mice and mice with heterozygous R2474S mutation in RyR2 (RyR2-R2474S) that exhibit exercise-induced ventricular arrhythmias and sudden cardiac death, first reported by Lehnart et al. [19]. We conducted dual dye optical mapping of transmembrane voltage (*V*_*m*_) and intracellular Ca^2+^ transients (CaT) in Langendorff perfused hearts, providing high-throughput characterization of action potentials (APs) and intracellular Ca^2+^ transients (CaT) and their relationship in hearts from WT and RyR2-R2474S mice under baseline and adrenergic stress conditions. We performed four series of detailed electrophysiological analyses in in vivo and intact hearts. Firstly, we assessed the arrhythmic incidence under basal and adrenergic stress conditions in wild-type control and RyR2^R2474S/+^ mice in vivo and ex vivo hearts. Then, we characterized the global and regional ventricular profiles of calcium transient properties and action potentials in WT and RyR2-R2474S hearts. This enabled us to determine how defective channels alter the calcium (C-) clock at the cellular level results in Ca^2+^ transients global and regional effects and determined whether such altered C-clock also results in an alteration of M-clock through alterations in AP depolarization or repolarization dynamics. Then we analyzed the conduction properties of V_m_ in Langendorff perfused hearts from WT and Ryr2-R2474S hearts, which allows us to understand how defective C-clock in mutant heart feeds back onto the M-clock, altering AP conduction. Finally, by analyzing voltage ‑calcium latency, we explored the C- and M-clock coupling and how defective RyR2-R2474S channels particular the Ca^2+^ alternans affect such coupling thus underlying arrhythmogenesis. Taken together, our study provides new insights into the arrhythmogenic mechanisms of CPVT at tissue level.

## 2 Materials and methods

### 2.1 Ethics and study approval

All procedures were approved by the Institutional Animals Ethics Committee at Southwest Medical University, Luzhou, China under the national guidelines under which the institution operates, which conform with the National Institutes of Health (NIH) Guide for the Care and Use of Laboratory Animals. Wild type (WT) and *RyR2*-R2474S/+ knock-in mice were provided by the Experimental Animal Center of Southwest Medical University and maintained in its pathogen−free facility with ad libitum access to food and water.

### 2.2 ECG monitoring

Mice (male and female, 8–10 weeks) were anesthetized with 2–5 % isoflurane using a gas anaesthesia machine (RWD Life Science, Shenzhen City, Guangdong Province, China). ECG monitoring before and through the 30 min following isoprenaline (ISO) (1 or 10 mg/kg i.p.; Cat: HY-B0468; MedChem Express, USA) challenge in both *RyR2*-R2474S/+ (*n* = 8) and WT mice (n = 8) used the multi‑lead MP150 recording instrument (BIOPAC system, Inc. USA).

### 2.3 Langendorff-perfused isolated hearts

C57 mice (male and female, 8–10 weeks) were anesthetized as described above. Hearts were quickly excised and placed in Krebs solution (mM: 128 NaCl, 20 NaHCO_3_, 4 KCl, 1.2 NaH_2_PO_4_, 1.05 MgCl_2_, 1.35 CaCl_2_, and 10 d-glucose, equilibrated with 5 % CO_2_ and 95 % O2; pH titrated to 7.4), then mounted onto a Langendorff perfusion system and perfused with Krebs solution with a 2 mL/min flow rate at 37 °C. Hearts were perfused and monitored for stability for 10 min before commencing experimental procedures.

### 2.4 Optical mapping of ex vivo cardiac preparations

After the Langendorff-perfused hearts reached steady state, contraction artefacts were minimized using blebbistatin (10 μM). Dye loading was aided by pre-perfusion with pluronic F127 (20 % *w*/*v* in DMSO). RH237 (1 mg/mL, stock solution Cat: S1109, Thermo Fisher Scientific, USA) and Rhod2-AM (1 mg/mL, stock solution, Cat: ab142780, Abcam, UK) were perfused to enable simultaneous membrane voltage (*V*_m_) and intracellular Ca^2+^ measurements at 37 °C. The detailed dye loading process were: after the contraction were minimized by blebbistatin, 15 μL stock solution of Rhod2-AM and 15 μL pluronic F127 were diluted in 20 mL Krebs solution and circulated in a total circulating volume of 50 ML for 15 mins, then 10 μL stock solution of RH237 was diluted in the 50 mL circulating Krebs solution. Once the dye loading is finished, hearts were electrophysiologically assessed with the optical mapping method, using a custom-designed optical mapping system equipped with an electron multiplying charge-coupled devices (EMCCD) camera (Evolve 512, Photometrics, Tucson, AZ, United States). Hearts were kept in Krebs solution containing 10 μM blebbistatin at 37 °C during imaging. Two 530 nm light emitting diodes (LEDs) were used for excitation of the Ca^2+^-sensitive dye Rhod-2 AM and voltage-sensitive dye RH237. CaT fluorescence was collected using 585/40 nm bandpass filter while Vm emission was collected using a 662 nm longpass filter. The high-speed EMCCD camera (Evolve 512, Photometrics, Tucson, AZ, United States), giving a high temporal resolution of sub-frames, up to 1000 frames/s, and minimum spatial resolution at the sample of 32 × 32 μm per pixel. The stimulator and ECG recording were simultaneously driven by Spik2 software. For the analysis of optical mapping signals and generation of isochronal maps, data were semi-automatically processed using a modified version of the ElectroMap and OmapScope5 software [20].

### 2.5 Arrhythmia induction protocol

Stimuli were generated by an isolated constant voltage/current stimulator (SEN7203, Nihon Kohden, Japan). They were delivered with a platinum electrode onto the epicardial apex at an amplitude 2 × the diastolic voltage threshold and a 2 ms pulse width. The optical mapping was performed in conjunction with an alternans mapping protocol. The heart was consecutively paced at basic cycle lengths (BCL) from 100 ms with progressive 10 ms decrements until a cycle length of 50 ms was reached. The heart was then paced with high frequency, 50 Hz, stimulation. The heart was paced 50 times with 2 ms duration pulses and the optical mapping recording was performed all the time in each episode. This protocol was performed both before and after ISO (1 μM) was delivered.

### 2.6 Statistical analysis

All data are presented as mean ± standard error the mean, with individual data points superimposed. Differences between group means were examined using two-tailed, paired Student's *t*-test or using two-way Analysis of Variance (ANOVA) with Sidak's test for multiple comparisons to a *P* < 0.05 significance level.

## 3 Results

### 3.1 RyR2-R2474S/+ hearts expressed a high pro-arrhythmic tendency

**Fig. 1:**
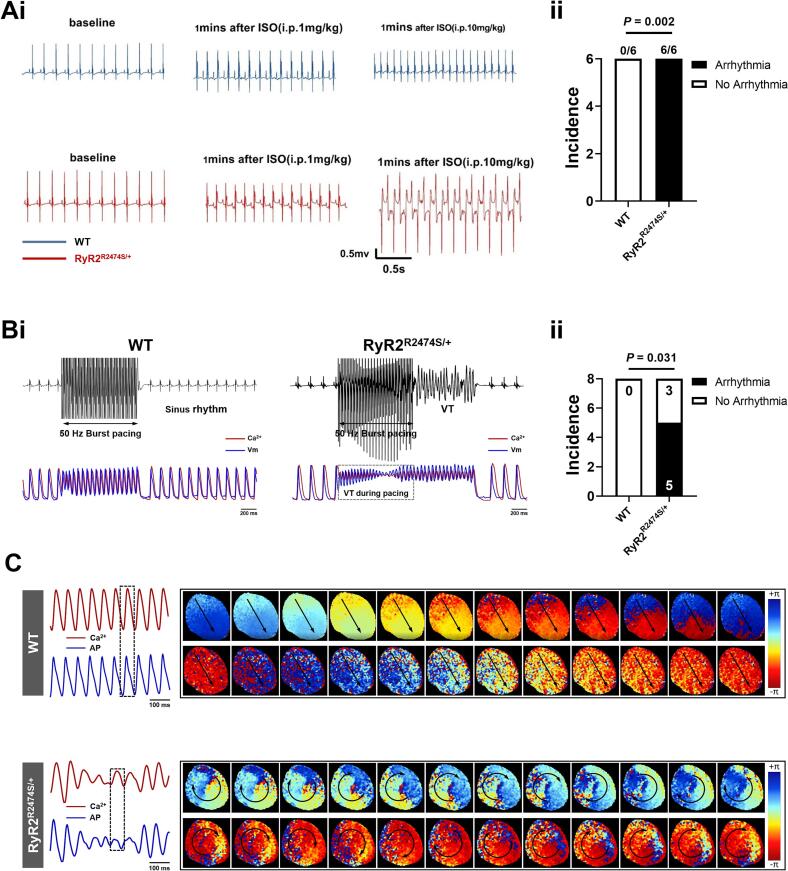
ECG monitoring and optical mapping recordings in WT and *RyR2*-R2474S/+ mice. (A) Representative in vivo ECG recordings (i) and incidence of cardiac arrhythmia (ii) in *RyR2*-R2474S/+ mice before and during ISO challenge (ISO, 10 mg/kg. WT: arrhythmias in 0/6 hearts, *RyR2*-R2474S/+: arrhythmias in 6/6 hearts). (B) Optical recordings of responses to ISO (1 μM) challenge (i) and incidence of cardiac arrhythmia (ii) in ex vivo WT and *RyR2*-R2474S/+ hearts (WT: arrhythmias in 0/8 hearts, *RyR2*-R2474S/+: arrhythmias in 5/8 hearts). (C) Typical optical mapping recording showing pro-arrhythmic re-entry patterns in *RyR2*-R2474S/+ hearts but not WT hearts.

We first assessed pro-arrhythmic tendency in WT and RyR2-R2474S/+ mice in vivo (ECG) and ex vivo (ECG, cell membrane voltage (*Vm*), and Ca^2+^ transients (CaT)) before and during adrenergic challenge. Isoprenaline (ISO; 10 mg/kg) challenge elicited a high incidence of ventricular arrhythmic events, here illustrated by a biventricular tachycardia (Fig. 1Ai), in *RyR2*-R2474S/+ but not WT mice in vivo (Fig. 1Aii, *P* < 0.05, *n* = 6 for each group) and ex vivo (Fig. 1Bii, *P* < 0.05, *n* = 8 for each group). Phase maps demonstrated abnormal re-entrant patterns associated with ventricular arrhythmias in overall cardiac electrical activity in *RyR2*-R2474S/+ hearts while WT hearts maintained normal conduction. In isolated hearts, typical polymorphic VT was observed in *RyR2*-R2474S/+ hearts during adrenergic challenge combined with fast pacing protocol (50 Hz). This occurs with multiple activation foci detected by voltage membrane potential mapping as illustrated in Fig. 1C.

### 3.2 *RyR2*-R2474S/+ hearts presented abnormal Ca^2+^ dynamics without accompanied action potential duration and conduction abnormality

**Fig. 2:**
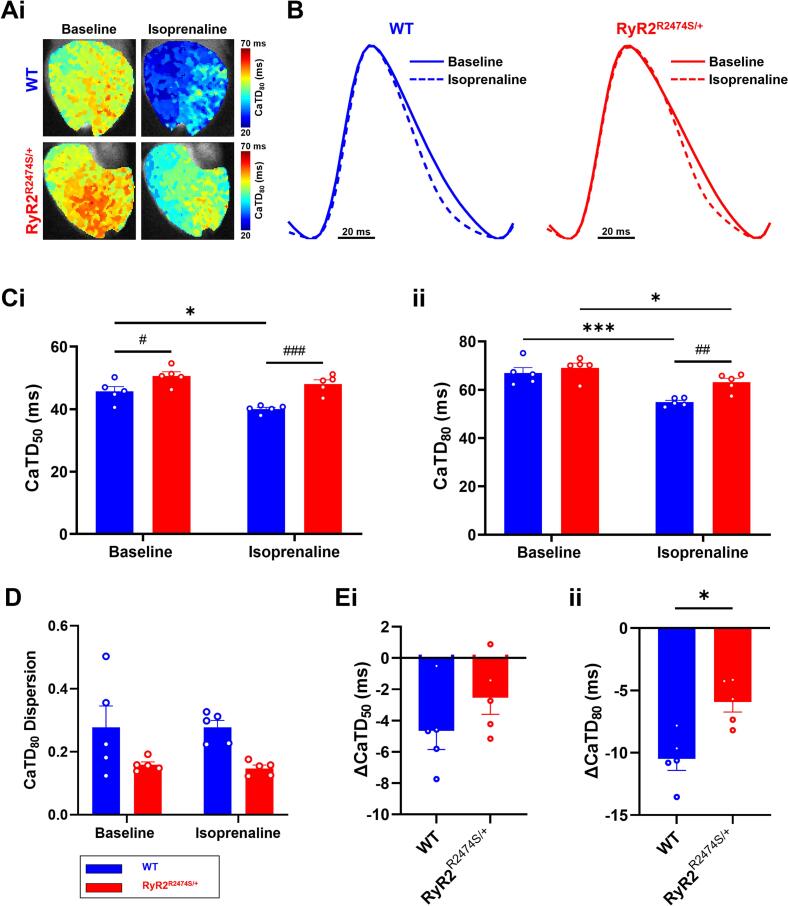
Calcium transient (CaT) properties of WT and *RyR2*-R2474S/+ hearts. Typical (A) CaT duration (CaTD) maps at 10 Hz. (B) Averaged optical CaT traces before and during ISO challenge. (C) Averaged CaTD_50_ (i) and CaTD_80_ (ii) and (D) CaTD_80_ dispersion before and during 1 μM ISO challenge. (E) Alterations in CaTD_50_ (ΔCaTD_50_, (i)) and CaTD_80_ (ΔCaTD_80_, (ii)) induced by 1 μM ISO challenge, in WT (*n* = 5) and *RyR2*-R2474S/+ hearts (n = 5). (*p < 0.05, **p < 0.01, Baseline vs. 1 μM ISO. ##p < 0.01, ###*p* < 0.001, WT vs *RyR2*-R2474S/+ mice). Two-way Analysis of Variance (ANOVA) with Sidak's test for multiple comparisons.

**Supplementary Fig. 1:**
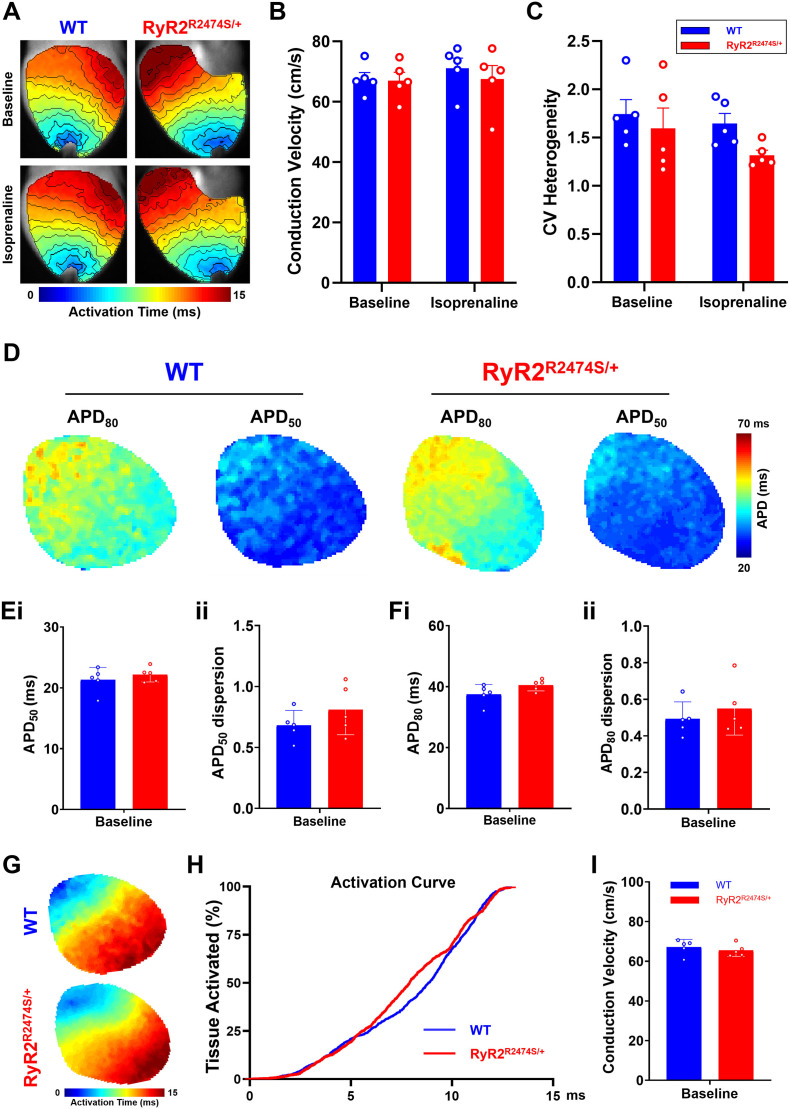
Action potential conduction properties in WT and *RyR2*-R2474S/+ hearts. (A) Typical isochronal maps. (B) Averaged conduction velocities and (C) conduction velocity dispersion before and following 1 μM ISO challenge in WT (*n* = 5) and *RyR2*-R2474S/+ hearts (n = 5). Panels D to I are based on voltage dye-only (RH237) optical mapping at baseline. (D) typical APD_80_ and APD_50_ maps of WT and *RyR2*-R2474S/+ hearts. (E) Statistical results of APD_50_ (i) and its dispersion (ii). (F) Statistical results of APD_80_ (i) and its dispersion (ii). Isochronal maps (G), activation curves (H) and CV statistical results (I) of WT and *RyR2*-R2474S/+ hearts. (n = 5 for each group).

The proarrhythmic phenotypes were then further investigated employing high-resolution dual-dye optical mapping of Ca^2+^ homeostasis and ventricular action potential activity in regularly paced WT and *RyR2*-R2474S/+ Langendorff perfused hearts. For excluding the influence of the results due to spectral overlap or physiological effects between voltage and calcium dyes, we conducted the mapping with single voltage dye or Ca^2+^ dye only. The results were similar to those obtained with dual dye loading (Supplementary Fig. 1). Fig. 2 illustrates results from assessing the durations of CaT traces following action potential stimulation. The CaT maps illustrate time to x = 50 % and 80 % return to baseline, CaTD_x._ The typical heatmap of CaTD_80_ was shown in Fig. 2A. Signals from the whole epicardial surface was averaged to yield overall CaT traces from both WT (blue traces) and *RyR2*-R2474S/+ ventricles (red traces) before (continuous traces) and during ISO (1 μM) challenge (dotted traces) (Fig. 2B). Dispersions in the measured parameters were measured as the spread between the 5th and 95th percentile of measurements across the heart, divided by the median value. The averaged CaTD_50_ and CaTD_80_ data together showed that *RyR2*-R2474S/+ showed significantly longer CaTD_50_ than WT both before and during ISO challenge (Fig. 2Ci and Cii). ISO challenge shortened the CaT transients in both experimental groups, but to smaller extents, ΔCaTD, in the *RyR2*-R2474S/+ than in the WT ventricles (Fig. 2Ei, Eii). Furthermore, the *RyR2*-R2474S/+ genotype showed reduced CaTD_80_ dispersion relative to WT and these were unchanged by ISO challenge (Fig. 2D).

**Fig. 3:**
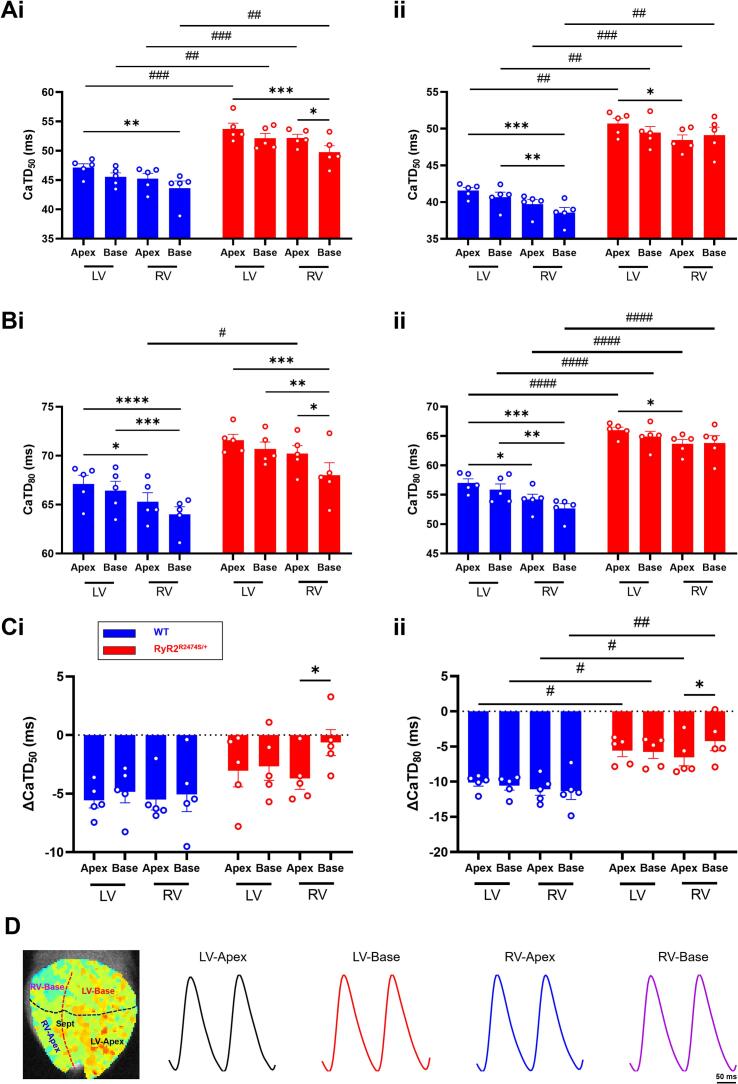
Regional calcium transient (CaT) properties in WT and *RyR2*-R2474S/+ hearts. (A) Regional CaTD_50_ and (B) CaTD_80_ (means ± SEM) before (i) and during 1 μM ISO challenge (ii) and (C) their resulting alterations, ΔCaTD_50_ (i) and ΔCaTD_80_ (ii) (means ± SEM), in WT (n = 5) and *RyR2*-R2474S/+ hearts (n = 5). (D) Typical CaTD_80_ heatmap and sketch map was shown for regional distribution analysis. LV = left ventricle. RV = right ventricle. *p < 0.05, **p < 0.01, ***p < 0.001, ****p < 0.0001. Two-way Analysis of Variance (ANOVA) with Sidak's test for multiple comparisons.

Systematic analysis of CaTD_50_ and CaTD_80_ at apical and basal left and right ventricular (LV and RV) regions of the *RyR2*-R2474S/+ and WT ventricles corroborated these trends. The local CaT readouts showed a trend towards the longest CaTD in the LV apex compared to all other regions both before and during ISO challenge in both populations. Before ISO challenge, in addition to some RV-LV differences (denoted by *); the region-by-region comparisons demonstrated systematically greater CaTD_50_ and CaTD_80_ in *RyR2*-R2474S/+ relative to WT (denoted by #) (Fig. 3Ai, Bi). ISO challenge accentuated these differences which now extended to both CaTD_50_ and CaTD_80_ (Fig. 3Aii and 3Bii). In consequence, ISO challenge produced a reduced shortening of CaT duration ΔCaTD_80_ in *RyR2*-R2474S/+ relative to WT. This is reflected in the significance levels observed in the LV apex and base, and RV apex (Fig. 3Ci and Cii). Fig. 3D showed the typical CaTD_80_ heatmap and sketch map for regional distribution analysis.

**Fig. 4:**
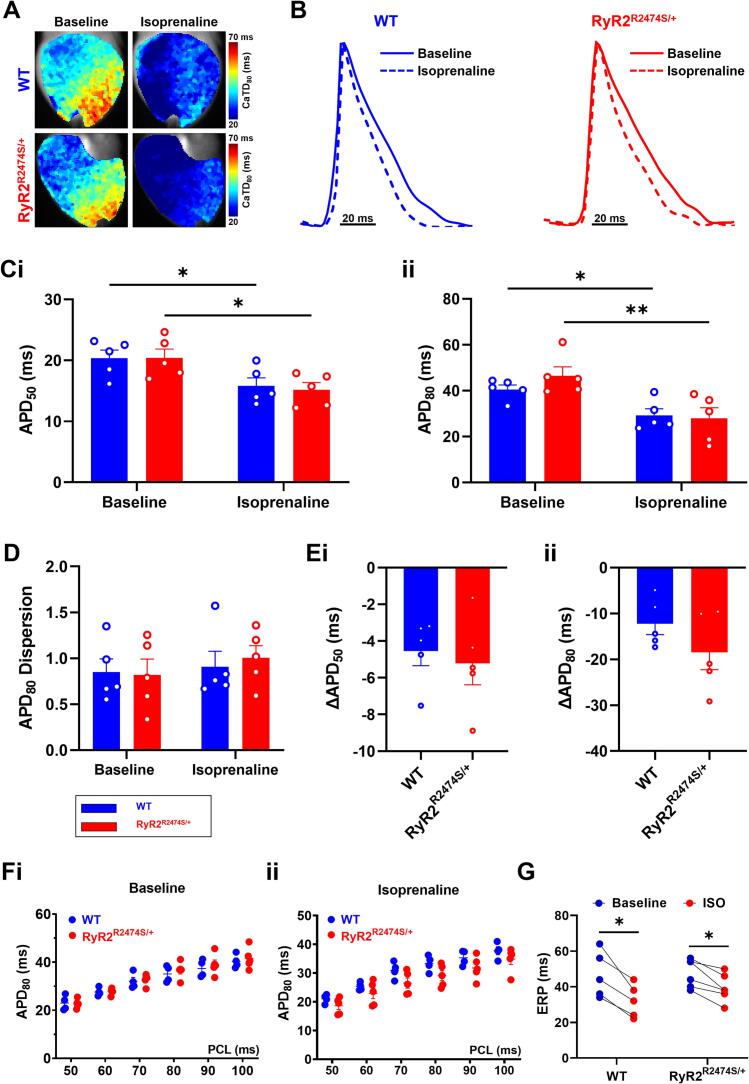
Action potential properties of WT and *RyR2*-R2474S/+ hearts. Typical maps of action potential duration at 80 % recovery (APD_80_) (A) and averaged optical action potential traces (B). (C) Averaged APD_50_ (i) and APD_80_ (ii) and (D) averaged APD_80_ dispersion before and during 1 μM ISO challenge. (E) Alterations in APD_50_ (ΔAPD_50_, (i)) and APD_80_ (ΔAPD_80_, (ii)) induced by 1 μM ISO in WT (n = 5) and *RyR2*-R2474S/+ hearts (n = 5). (F) Restitution curves and ERP (G) between *RyR2*-R2474S/+ and WT heart with or without challenge of ISO (F) (n = 5 for WT, n = 5 for *RyR2*-R2474S/+ group). *p < 0.05, **p < 0.01. Two-way Analysis of Variance (ANOVA) with Sidak's test for multiple comparisons.

The parallel electrophysiological *V*_m_ measurements revealed ventricular action potential durations (APD_x_) at either x = 50 % or 80 % full recovery, APD_50_ and APD_80_. Their dispersions and action potential propagation readouts were contrastingly either unchanged or altered in directions contrasting with those of the CaT results. Fig. 4 illustrates the related (A) APD_80_ maps and (B) typical averaged action potential waveforms, and APD_50_ (Ci) and APD_80_ values (Cii). These were indistinguishable between *RyR2*-R2474S/+ and WT hearts before or during ISO challenge. Both *RyR2*-R2474S/+ and WT showed similar but significantly lower values with ISO challenge (C, E). The APD_80_ dispersions remained indistinguishable through both cardiac genotypes and pharmacological conditions. In addition, we further investigated the restitution curves with S1S1 pacing and ERP with S1S2 pacing. The data shown in Fig. 4F and G demonstrated that there was no significant difference about the restitution curves and ERP between *RyR2*-R2474S/+ and WT heart with or without ISO challenge.

**Fig. 5:**
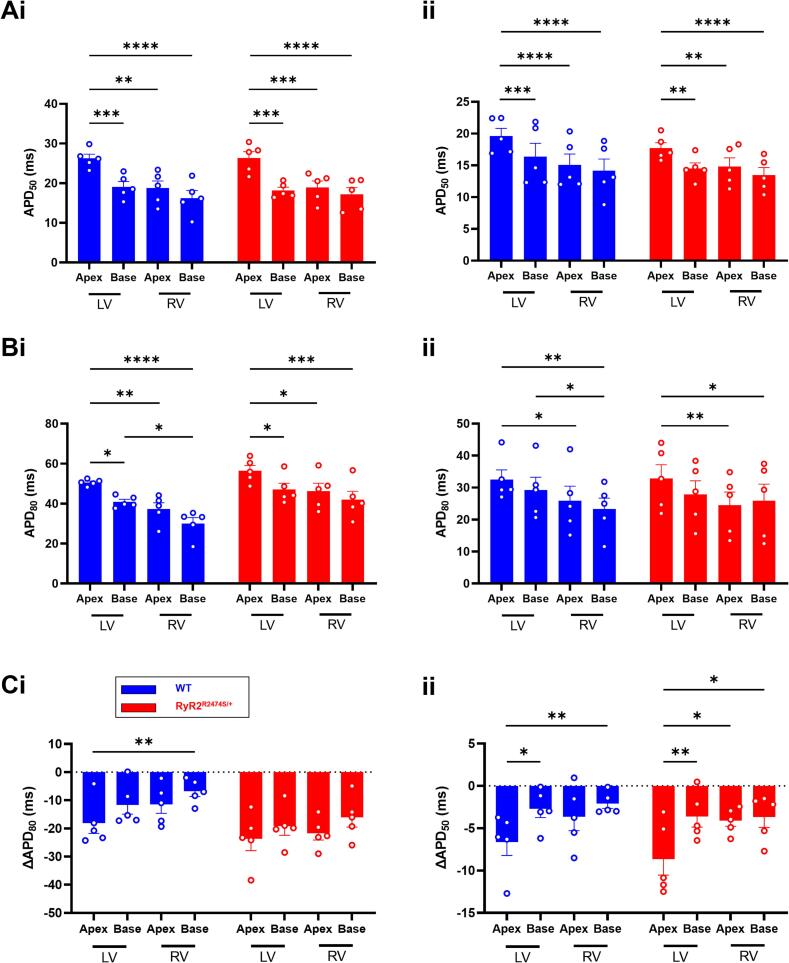
Regional action potential properties of WT and *RyR2*-R2474S/+ hearts. (A) Regional APD_50_ and (B) APD_80_ values before (i) and during 1 μM ISO challenge (ii) and (C) the resulting ΔAPD_50_, (i) and ΔAPD_80_ (ii) in WT (n = 5) and *RyR2*-R2474S/+ hearts (n = 5). LV = left ventricle. RV = right ventricle. *p < 0.05, **p < 0.01, ***p < 0.001, ****p < 0.0001. Two-way Analysis of Variance (ANOVA) with Sidak's test for multiple comparisons.

Systematic analyses of APD_50_ and APD_80_ through the different apical and basal, LV and RV, regions in WT and *RyR2*-R2474S/+ hearts before and during ISO challenge confirmed this overall trend. Here, the LV apex showed the longest APD_50_ and APD_80_ amongst ventricular regions whether before or during the ISO challenge (Fig. 5A and B). There did exist significant apex-base, LV-RV, APD_50_ and APD_80_, differences within each WT or *RyR2*-R2474S/+ group both before and during the ISO challenge. However, there were no differences in these readouts between corresponding recording sites in the WT or *RyR2*-R2474S/+, whether before or during the ISO challenge in any given region. Thus, ISO challenge produced similar shortenings, ΔAPD_50_ and ΔAPD_80_, through all regions in both WT and *RyR2*-R2474S/+ (Fig. 5C). There was no difference in dispersion in the APD_80_ readings between groups or conditions. Measurements of ventricular conduction velocity (Supplementary Fig. 1) demonstrated similar velocities in both WT and *RyR2*-R2474S/+ hearts under both baseline and ISO challenge conditions (Supplementary Fig. 1A and B), with dispersions indistinguishable between these (Supplementary Fig. 1C).

### 3.3 *RyR2*-R2474S/+ hearts present abnormal temporal relationships between the CaT and *V*_m_

**Fig. 6:**
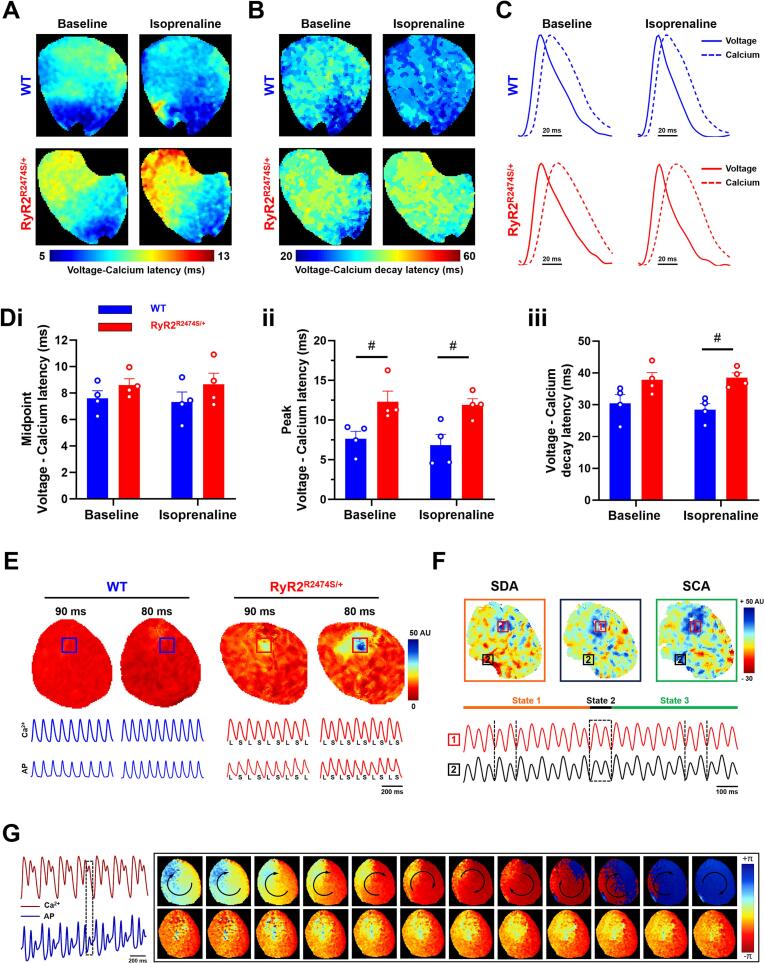
Comparisons of CaT and Vm findings in WT and *RyR2*-R2474S/+ hearts and CaT abnormalities during ISO challenge in *RyR2*-R2474S/+ hearts. Typical voltage‑calcium latency mapping of (A) half rise time and (B) time to 50 % full recovery and averaged dual voltage‑calcium signals. (C) Averaged time lags between their half rise time, (i), time to peak (ii) and time to half recovery (iii) before and during 1 μM ISO challenge in WT (*n* = 4) and *RyR2*-R2474S/+ hearts (n = 4). (D) frequency-dependent CaT alternans was observed in *RyR2*-R2474S/+ hearts after ISO challenge, not in WT mice. Changeable CaT alternans manifested as SDA and SCA (E) and phase maps showing reentrant activity (F) under 50 Hz burst pacing after ISO challenge in *RyR2*-R2474S/+ hearts. #p < 0.05, ##p < 0.01 WT vs. *RyR2-*R2474S/+ mice. Two-way Analysis of Variance (ANOVA) with Sidak's test for multiple comparisons.

**Fig. 7:**
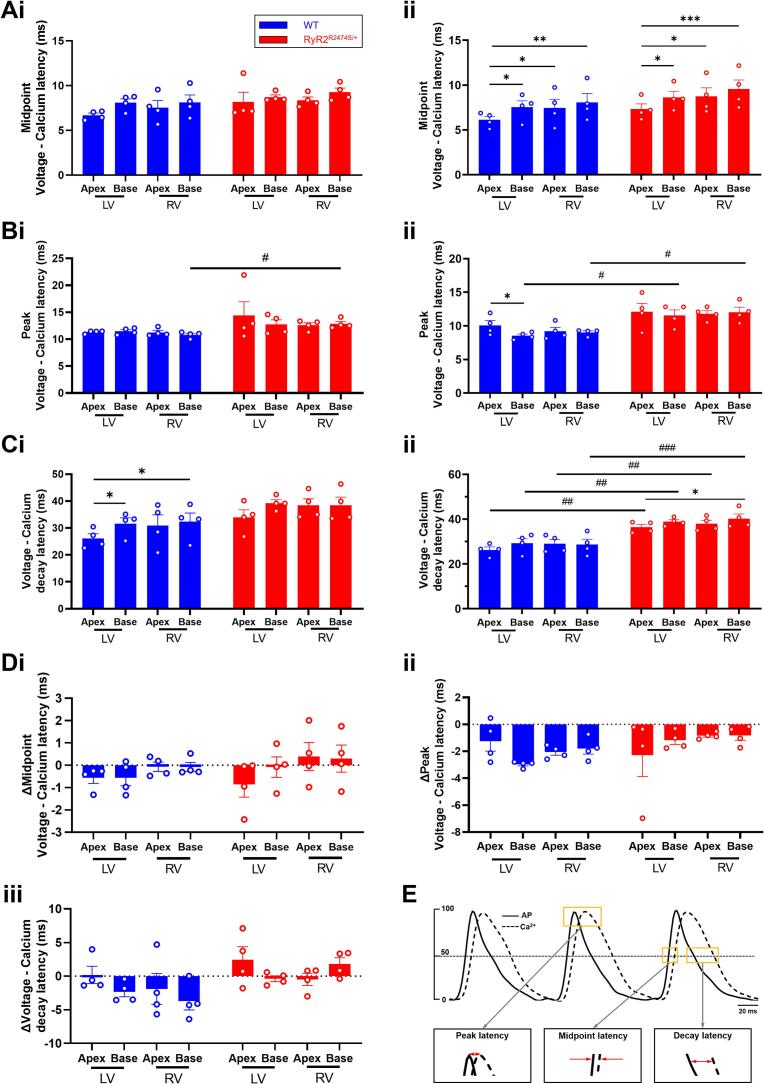
Regional voltage-calcium properties in WT and *RyR2*-R2474S/+ hearts. Regional latencies between (A) half rise time, (B) time to peak and (C) time to half recovery before (i) and following 1 μM ISO challenge (ii), and (D) the resulting changes in latencies between half rise time (i), time to peak (ii) and time to half recovery (iii), in WT (n = 4) and *RyR2*-R2474S/+ mice (n = 4). (E) Schematic diagram of voltage‑calcium latency. LV = left ventricle. RV = right ventricle. *p < 0.05, **p < 0.01, ***p < 0.001. Two-way Analysis of Variance (ANOVA) with Sidak's test for multiple comparisons.

The final analyses compared temporal relationships between the CaT and *V*_m_ signal waveforms obtained under the different conditions in the experimental groups. The *V*_m_-CaT latencies were quantified by the time intervals between their respective rising time to half peak, their time to peak and their time to half decay. Results from *RyR2*-R2474S/+ and WT hearts before and following ISO challenge were compared. Fig. 6A and B exemplifies the latencies for the upstroke and decay phases under these conditions. Fig. 6C illustrates the corresponding averaged and superimposed CaT and *V*_m_ traces. These demonstrated similar *V*_m_-CaT latency involving rising time to half peak time, and divergent *V*_m_-CaT latency in their time to peak in *RyR2*-R2474S/+ compared to WT (Fig. 6Di, Dii) *RyR2*-R2474S/+ hearts demonstrated a significantly prolonged latency time between peak transmembrane voltage and peak cytosolic Ca^2+^, and time to 50 % voltage repolarization and decay in cytosolic Ca^2+^ (Fig. 6Diii). As shown in Fig. 6E, after ISO intervention under S1S1 programmed pacing, *RyR2*-R2474S/+ hearts exhibited frequency-dependent CaT alternans and corresponding APD alternans but not WT hearts. Such CaT and APD alternans occurred mostly semiannually in specific region of the ventricular pre occurrence of VT. Interestingly, following 50 Hz burst pacing in a *RyR2*-R2474S/+ heart, ventricular localized spatial discordant CaT alternans were observed and then quickly converted into spatial concordant alternans (Fig. 6F). Moreover, though phase analysis, we found that CaT oscillation manifested as rotating waves (Fig. 6G). Fig. 7 illustrated systematic statistical results of regional analysis of voltage‑calcium latencies. Fig. 7Ai showed that no difference of midpoint latencies in all region of WT and *RyR2*-R2474S/+ hearts, while ISO intervention broke their regional balance (Fig. 7Aii). RV peak latencies in *RyR2*-R2474S/+ hearts were prolonged compared with WT hearts (Fig. 7B). Increased decay latencies were observed in *RyR2*-R2474S/+ relative to WT hearts during ISO challenge (Fig. 7C). Fig. 7D showed no difference was found in the Δ value of latency alteration in both groups. The scheme diagram of latencies was illustrated in Fig. 7E.

## 4 Discussion

Of genetic abnormalities implicated in CPVT, those involving RYR have the commonest clinical incidence. The RYR mutations associated with CPVT cluster in 3 disease-susceptible regions of the channel [21]. Those in the central RYR2 region include the *RyR2*-R2474S missense mutation first reported in identical twin brothers presenting with syncope and exercise-induced ventricular tachycardia (VT) [6]. Subsequent studies associated *RYR2*-R2474S with increased sensitivity to PKA resulting in increased open probabilities and gating frequencies [7]. This increased sensitivity to Ca^2+^-induced activation occurred even at moderate [Ca^2+^]_i_. Heterozygous, *RyR2*-R2474S/+, mice exhibited exercise-induced ventricular arrhythmias and sudden cardiac death recapitulating clinical CPVT [19]. They also showed spontaneous generalized tonic-clonic seizures that could occur in the absence of cardiac arrhythmias. Treatment with the *RyR2*-specific agent S107 that enhances calstabin2 binding to *RyR2*-R2474S channel inhibited the channel leak, prevented the cardiac arrhythmias, and raised the seizure threshold. Interestingly, two studies recently reported that long-term exercise training reduced the arrhythmic propensity while restoring normal Ca^2+^ handling in *RyR2*-R2474S mice [22,23].

However, to date, to the best of our knowledge, there are no reports clarifying the relationship between transmembrane potential changes (*V*_m_) accompanying the action potential and intracellular Ca_2+_ transients (CaT) during excitation in intact murine *RyR2*-R2474S hearts. The present characterization first demonstrated an increased arrhythmogenic tendency together with abnormal Ca^2+^ transients in *RyR2*-R2474S/+ hearts particularly during the ISO challenge. Phase maps showed accompanying abnormal re-entry patterns associated with ventricular arrhythmias. Our murine *RyR2*-R2474S platform thus recapitulated the exercise-induced ventricular tachycardia phenotypes previously reported in such patients [6].

Detailed ventricular epicardial mapping then determined both overall averaged CaT characteristics and localized readouts from individual corresponding apical and basal LV and RV epicardial regions. The LV apex demonstrated the greatest CaT durations (CaTD). However, all recording sites in unchallenged *RyR2*-R2474S/+ showed longer CaTD than the corresponding regions in WT hearts. ISO (1 μM) challenge then shortened the CaTD but accentuated the differences between genotypic groups. The latter suggests that ISO reduced CaTD less effectively in *RyR2*-R2474S/+ than WT. *V*_m_ mapping similarly showed the longest APD_50_ and APD_80_ in the LV apical regions, and apex-base and LV-RV differences within both *RyR2*-R2474S/+ and WT groups both before and during ISO stress. However, unchallenged *RyR2*-R2474S/+ and WT showed similar APD_50_ and APD_80_. ISO challenge then shortened both APD_50_ and APD_80_ but with similar effectiveness in *RyR2*-R2474S/+ and WT which continued to show similar APD_50_ and APD_80_. *RyR2*-R2474S/+ and WT, whether before or during ISO challenge, showed similar action potential conduction velocities.

These contrasting CaT and *V*_m_ changes are compatible with altered excitation contraction coupling processes distinct from any accompanying membrane excitation changes. The differences were quantified by deriving and comparing differences between the corresponding CaT and *V*_m_ traces. We systematically determined *V*_m_-CaT latency between their rising time to half peak, time to peak and time to half recovery between the different ventricular epicardial regions. Differences in such *V*_m_-CaT latency between *RyR2*-R2474S/+ and WT hearts before and during ISO challenge occurred for the time to half recovery. Such findings thus selectively implicate a delayed inactivation in CaT relative to action potential recovery in the pro-arrhythmic action of *RyR2*-R2474S/+ in intact hearts.

Ca^2+^ or/and APD alternans were the risk factor for life threatening cardiac arrhythmias and the important underlying mechanism of occurrence of cardiac arrhythmia [24]. APD alternans lead to larger wavelength oscillations and heterogeneity, which may contribute to electrical instability, isotopic beat and reentry, which accelerated the occurrence of ventricular arrhythmias [25]. APD alternans occurred with or without CaT alternans. In this study, abnormal Ca^2+^ transients were the original initiator of CPVT in *RyR2*-R2474S/+ mice. Therefore, APD alternans always occurred with CaT alternans. It was interesting that CaT alternans showed obvious heterogeneity (Fig. 6E). The localized CaT alternans may be the important original trigger of CPVT in *RyR2*-R2474S/+.

In conclusion, our mapping study of the transmembrane potential (*Vm*) and intracellular Ca^2+^ transient (CaT) in intact hearts of *RyR2*-R2474S mice, in particular the analysis of voltage-calcium latency, provide new insights into the arrhythmogenic mechanisms of CPVT at tissue level.

The following is the supplementary data related to this article.

## CRediT authorship contribution statement

**Yangpeng Li:** Writing – original draft, Investigation, Formal analysis, Data curation. **Zhu Liu:** Methodology, Formal analysis, Data curation. **Christopher O'Shea:** Writing – review & editing, Software, Methodology. **Jianhong Li:** Software, Methodology, Investigation, Data curation. **Xian Luo:** Formal analysis, Data curation. **Tangting Chen:** Writing – review & editing, Formal analysis, Data curation. **Xianhong Ou:** Software, Data curation. **Weichao Liu:** Software, Formal analysis. **Guoliang Hao:** Software. **Christopher L.-H. Huang:** Writing – review & editing. **Davor Pavlovic:** Writing – review & editing. **Xiaoqiu Tan:** Writing – original draft, Supervision, Funding acquisition, Conceptualization. **Ming Lei:** Supervision, Conceptualization.

## Funding

This research was funded by the 10.13039/501100001809National Natural Science Foundation of China [No. 82270334 (XT), 81670310 (XT), No. 81700308 (XO) and No. 31871181 (ML)], 10.13039/501100004829Science and Technology Department of Sichuan Province, China [No. 2022YFS0635 (YL), No. 2024JDHJ0051 (XT), No. 2022YF0607 (XT)] and the 10.13039/501100000274British Heart Foundation, UK [PG/14/80/31106 (ML), PG/16/67/32340 (ML), PG/19/59/34582 (CLHH), PG/14/79/31102 (CLHH)]. COS: 10.13039/100010269Wellcome (221650/Z/20/Z), BHF (AA/18/2/34218), 10.13039/501100000266EPSRC (L016346). DP: Wellcome (109604/Z/15/Z), BHF (PG/17/55/33087, RG/17/15/33106, FS/19/12/34204, FS/19/16/34169), EPSRC (L016346).

## Declaration of competing interest

The authors declare that the research was conducted in the absence of any commercial or financial relationships that could be construed as a potential conflict of interest.
